# Prevalence and risk factors of diabetes mellitus and hypertension in North East Tunisia calling for efficient and effective actions

**DOI:** 10.1038/s41598-023-39197-0

**Published:** 2023-08-05

**Authors:** Nadia Kheriji, Thouraya Dakhlaoui, Wafa Kamoun Rebai, Sonia Maatoug, Mohamed Taher Thabet, Thouraya Mellah, Mehdi Mrad, Hajer Trabelsi, Manel Soltani, Maria Kabbage, Hichem Ben Hassine, Afef Hadj Salah Bahlous, Faten Mahjoub, Henda Jamoussi, Abdelmajid Abid, Sonia Abdelhak, Rym Kefi

**Affiliations:** 1 Laboratory of Biomedical Genomics and Oncogenetics, Institut Pasteur de Tunis, 13, Place Pasteur, Belvédère Tunisie, B.P. 74, 1002 Tunis, Tunisia; 2 University of Tunis El Manar, Tunis, Tunisia; 3 Faculty of Medicine of Tunis, Tunis, Tunisia; 4 Regional Association of Diabetics of Zaghouan-Regional Hospital of Zaghouan, Zaghouan, Tunisia; 5 Science Shop (Communication, Science and Society Unit)“Science Together-العلم مع بعضنا”, Institut Pasteur de Tunis, Tunis, Tunisia; 6 Higher School of Digital Economy (ESEN-UMA), University of Manouba, Manouba, Tunisia; 7 Association La Recherche en Action (REACT), Tunis, Tunisia; 8 Laboratory of Clinical Biochemistry and Hormonology, Institut Pasteur de Tunis, Tunis, Tunisia; 9 National Institute of Nutrition & Food Technology of Tunis, Tunis, Tunisia; 10 Research Unit UR18ES01 on “Obesity”, Faculty of Medicine of Tunis, Tunis, Tunisia

**Keywords:** Biochemistry, Cardiology, Diseases, Endocrinology, Health care, Medical research

## Abstract

Diabetes and hypertension are a serious public health problem worldwide. In the last decades, prevalence of these two metabolic diseases has dramatically increased in the Middle East and North Africa region, especially in Tunisia. This study aimed to determine the prevalence of type 2 diabetes (T2D) and High Blood Pressure (HBP) in Zaghouan, a North-East region of Tunisia. To this end, an exploratory study with stratified random sampling of 420 participants has been carried out. Various data were collected. Blood samples and urine were drawn for biochemical assay. Then, all data were analyzed using the statistical R software. Results showed an alarming situation with an inter-regional difference in prevalence of obesity (50.0%, CI 95.0%), HBP (39.0%, CI 95.0%) and T2D (32.0%, CI 95.0%). This study allowed the discovery of 24, 17 and 2 new cases of T2D, HBP and T2D&HBP respectively. The association of some socio-economic factors and biochemical parameters with these chronic diseases has been highlighted. To conclude, the health situation in the governorate of Zaghouan requires urgent interventions to better manage the growing epidemic of non-communicable diseases (NCD) in the region. This study demonstrated the importance of engaging health policy makers in road mapping and implementing national NCD prevention programs.

## Introduction

Type 2 diabetes (T2D) and High Blood Pressure (HBP) are a real burden that threaten lives considering the steep rise of their prevalence. The World Health Organization (WHO) define diabetes as a state of chronic hyperglycemia that results in fasting blood glucose (FBG) ≥ 7.0 mmol/l. This serious disease occurs when the pancreas does not produce enough insulin, or when the body becomes unable to effectively use the insulin. Insulin is a hormone that regulates the level of glucose in the blood, which is used to produce energy (https://www.who.int/news-room/fact-sheets/detail/diabetes).

Hypertension is defined when blood pressure is too high. Two numbers are used to represent blood pressure: The systolic value that represents the pressure in blood vessels when the heart contracts or beats and the diastolic value that represents the pressure in the vessels when the heart rests between beats. Hypertension is diagnosed if, when it is measured on two different days, the systolic blood pressure (SBP) readings on both days is ≥ 140 mmHg and/or the diastolic blood pressure (DBP) readings on both days is ≥ 90 mmHg (https://www.who.int/news-room/fact-sheets/detail/hypertension). In fact, these two metabolic diseases can lead to degenerative complications in eyes, blood vessels, heart and kidneys causing serious damages and deaths^1,2^. According to the 10th edition of International Diabetes Federation (IDF), 796,000 deaths were due to diabetes in 2021, which means one death every 5 s. Around 7.5 million deaths worldwide occur due to hypertension^3^. In 2021, 537 million people had diabetes^3^. The World Health Organization (WHO) reported that more than one billion people worldwide had hypertension^4^. The prevalence of these chronic diseases varies across regions and country income groups. Indeed, the Middle East and North Africa (MENA) region have the highest prevalence of hypertension and the second world’s highest rate of rise in diabetes prevalence. An epidemiological study conducted in patients from Algeria, Tunisia and Morocco showed an overall crude hypertension prevalence of 45.4%^5^. Hence, one of the objectives all around the world was to reduce the dramatic increase of these two multifactorial diseases. Since 2010, several public health systems have been working on reducing the prevalence of hypertension through the foundation of a doable and viable program^6^.

In Tunisia, the prevalence of diabetes and hypertension was at 23.0% in 2019^7^ and 47.4% in 2018 respectively^8^. A projection study estimated that the prevalence of T2D in Tunisia could reach 26.6% in 2027^9^. Most alarming, more than half of diabetic patients were undiagnosed^10^ and only 37.1% of people with HBP were under control. These high rates in prevalence and uncontrolled cases of diabetes and hypertension may be explained by few factors, including quick urbanization and civilization^11^ and insufficient screening of these diseases especially in rural areas with limited access to health care facilities. In this context, collaboration has been established between the Regional Association of Diabetics of Zaghouan, a “Tunisian Civil Society Organization (CSO)” and the Laboratory of Biomedical Genomics and Oncogenetics in Institut Pasteur de Tunis (IPT), a public health and research institution, to obtain basic data and insight on estimated prevalence of T2D and HBP in Zaghouan and identify the different clinical and environmental factors associated with both diseases. This multi-stakeholder project has put in collaboration different experts in epidemiology, statistics, and biochemistry. This study might draw the attention of health decision makers and advocates to the alarming health situation in the governorate of Zaghouan specifically and the whole country globally.

## Materials and methods

### Sampling method and data collection

This study was conducted in the frame of a collaboration between the “Regional Association of Diabetics of Zaghouan” and the Laboratory of Biomedical Genomics and Oncogenetics in IPT. In fact, during its raising awareness campaigns in the six districts of Zaghouan, a North-Eastern region of Tunisia, the CSO has noted high prevalence of T2D and HBP in the region. Taking this observation into account, a study of the prevalence of T2D and HBP in the governorate of Zaghouan as well as the determination of associated risk factors was proposed. Hence, an exploratory study has been conducted to investigate the health situation in the region of Zaghouan.

The study population were recruited using a stratified random sampling method^12^. The sample size was determined based on the size of the Zaghouan population (inhabitants’ number) that hosts 160,963 residents in 2018^13^. We used Cochran’s formula to set the size of the representative sample with a margin of error of 5.0% as the following^14^.

$${\text{Sample}}\;{\text{Size}}({\text{SS}}) = (1.96^{2} (0.5) \, \times (1 - 0.5))/0.05^{2} = 384\;{\text{Zaghouan}}\;{\text{adults}}.$$

- 1.96: sampling confidence interval, for CI 95.0%
- 0.5: expected proportion (p) of a population response or actual proportion. By default, it is set to 0.5, which allows for the largest possible sample
- 0.05: margin of sampling error, 5.0%

Thus, according to the formula, the sample size was around 384 Zaghouan adults. In practice, we increased the sample size to 420 volunteer participants from the six districts of Zaghouan distributed as mentioned in Table 1. The sample size was increased by 10.0% to ensure the required number of correct responses and measurement error.

**Table 1:** Sampling method used to study the Zaghouan population.

District name	Number of inhabitants 2018	Sample size required^a^	Sample size collected
Zaghouan	34,367	87	133
Ezzriba	20,765	53	80
BirMchergua	21,508	53	67
Fahs	43,678	105	97
Nadhour	28,550	59	29
Saouef	12,095	27	14
Total	160,963	383	420

^a^(Number of inhabitants_**(district)**_/Size of Zaghouan population) × 384; 384 is the sample size calculated by Cochran’s formula with a margin of error of 5.0%; The size of Zaghouan population is 160,963.

We included in the study all aboriginal inhabitants of the Zaghouan region aged of 30 years old or above whatever their health status (T2D/No T2D and/or HBP/No HBP). Excluded from the study were all non-inhabitants of the Zaghouan region and all participants with T1D. This study was driven during 2 months from June to August of 2020.

### Ethics approval

The study was conducted according to the declaration of Helsinki and was approved by the Ethical Committee of the Institut Pasteur de Tunis (Registration number IRB00005445, FWA00010074, ref .2019/14/I/LR16IPT/V4).

### Methods and variables of measurements

In the present study, many variables and measurements were studied such as demographic (gender, age, marital status), anthropometric (Body Mass Index (BMI), Waist circumference (WC)), clinical (medical history, diabetes history, Blood Pressure (BP), treatment), genealogical, socio-economic data, data related to life-style and biological variables (biochemical measures). Diabetes was defined as Fasting Plasma Glucose (FPG) ≥ 1.26 g/l (7.00 mmol/l) or HbA1C ≥ 6.5% in accordance to the American Diabetes Association (ADA) recommendations^15^. HbA1C was performed in the biochemistry laboratory in IPT using certified and standardized method. Screening for prediabetes and risk for future diabetes was based on the value of HbA1c (%). In this study, were considered as prediabetic, participants who were not previously diagnosed as diabetic with HbA1c ranges between 5.7 and 6.49%^15^. Hypertension was defined as systolic blood pressure (SBP) ≥ 140 mmHg and diastolic blood pressure (DBP) ≥ 90 mmHg at the time of the study^16^. Indeed, for participants who had high blood pressure at the moment of the recruitment and known as not hypertensive, they will be referred to a health clinic unit to measure their blood pressure for 1 week in order to confirm the diagnosis of HBP. In this study, were considered as pre-hypertensive, participants who had a SBP in the range of 120–139 mmHg and a DBP between 80 and 89 mmHg at the time of the study and known as not hypertensive subjects. Overweight and obesity were defined as BMI ranges of 25–29.9 kg/m^2^ and ≥ 30 kg/m^2^ respectively^17^. In this study abdominal overweight was defined as Waist Circumference (WC) ranged between 95 to 102 cm for men and from 81 to 88 cm for women. Abdominal obesity was considered present in men and women if WC was ≥ 102 and WC ≥ 88 cm^18^ respectively. WC was measured across the belly button wearing light clothing by trained staff using a measuring tape on the subject while they were standing and breathing normally.

A signed informed consent was obtained from the 420 participants recruited during the raising awareness campaigns organized by the CSO’s volunteers, who have been trained on good practice in data collection and participants recruitment, in the six districts of Zaghouan. Then, clinical examination and questionnaire were used to collect demographic (e.g., age, gender, place of residence in Zaghouan), anthropometric (e.g., height, weight), clinical (e.g.: blood pressure (BP), capillary blood glucose), genealogical, and socio-economic data (e.g. educational level, socio-professional category), as well as data related to life-style (physical activity, smoking, alcohol consumption and stress). Blood samples and urine were also collected from each participant. Finally, all samples were pseudonymised and centralized at the regional hospital of Zaghouan for biochemical analyses. Some biochemical analyses were performed in the biochemistry laboratory of in IPT. The biochemical parameters measured were: Fasting Plasma Glucose (FPG), Glycated Hemoglobin (HbA1c), Total Cholesterol (T-CHL), LDL-c (Low Density Lipoprotein), Triglycerides (TG), Albuminuria (Alb), High Sensitivity C Reactive Protein (CRP), urea, 25-hydroxy vitamin D (25-OH Vit D) and Creatinine. All clinical and biological data were centralized in a codified and secure database for statistical analyses.

### Statistical analyses

All collected data were integrated into a secure database for statistical analysis using the data mining R software for Windows version 4.0.2^19^. To describe the demographic and clinical characteristics of the population, means and frequencies (in percentages) for people with and without T2D and HBP were reported. Our population study was stratified into six groups according to the HbA1C level and the BP. A multivariate logistic regression analyses was conducted between two groups (NoT2D No HBP Vs T2D&HBP) to identify variables associated with both conditions. Statistical significance was set at P < 0.05. Comparison between groups was performed using Student t-test and chi2-test.

## Results

There were 420 eligible subjects who participated in this study. Table 2 describes all collected data: age distribution, BMI, WC and biochemical measurements among the study population. The mean age of participants was about 57.22 ± 13.52 years. Among these participants, 76.0% (n = 318) were female and 24.0% (n = 102) were male. The mean BMI of the study population was elevated in the range of 30.06 ± 5.88 kg/m^2^.

**Table 2:** Baseline characteristics of the study participants.

Characteristic	Mean ± SD	Min	Max
Age (years)	57.22 ± 13.52	30.00	88.00
BMI (kg/m^2^)	30.06 ± 5.88	14.80	55.74
WC (cm)	♀: 101.08 ± 13.00	♀: 65	♀: 162
	♂:101.00 ± 13.25	♂: 52	♂: 129
Capillary blood glucose (g/l)	1.35 ± 0.71	0.74	5.60
FPG (mmol/l)	6.50 ± 4.68	0.28	36.97
HbA1c (%)	6.58 ± 1.70	3.02	14.64
T-CHL (mmol/l)	5.05 ± 1.03	2.62	9.35
TG (mmol/l)	1.47 ± 0.82	0.48	6.79
LDL-c (mmol/l)	3.87 ± 1.48	1.21	20.59
Albuminuria (mg/l)	12.97 ± 29.45	2.54	257.36
25-OH Vit D (ng/ml)	19.80 ± 8.76	8.90	62.16
CRP (mg/l)	4.13 ± 9.87	0.00	113.08
Creatinine (µmol/l)	64.60 ± 19.90	34.00	117.30
Urea (mmol/l)	5.24 ± 2.07	0.48	26.35
SBP (mm Hg)	126.3 ± 22.9	80.00	220
DBP (mm Hg)	75.65 ± 12.47	40.00	122

*SD* standard deviation, *BMI (kg/m*^*2*^*)* body mass index, *WC (cm)* waist circumference, *PFG (mmol/l)* plasma fasting glucose, *HbA1c (%)* glycated hemoglobin, *T-CHL (mmol/l)* total cholesterol, *TG (mmol/l)* triglycerides, *LDL-c (mmol/l)* low density lipoprotein, *25-OH Vit D (ng/ml)* 25-hydroxy vitamin D, *CRP (mg/l)* C-reactive-protein, *SBP (mmHg)* systolic blood pressure, *DBP(mmHg)* diastolic blood pressure.

A proportion of 44.5% of men (n = 45) and most women of the study, 85.0% (n = 271) had abdominal obesity. Biochemical measures, diabetes and hypertension status for men and women with abdominal overweight and obesity are mentioned in Table 3.

**Table 3:** The biochemical profile of participants with abdominal overweight and obesity.

	Men		Women	
	Abdominal overweight N = 23 (23.0%) Mean ± SD	Abdominal obesity N = 45 (45.0%) Mean ± SD	Abdominal overweight N = 20 (6.0%) Mean ± SD	Abdominal obesity N = 271 (85.0%) Mean ± SD
BMI (kg/m^2^)	26.88 ± 2.44	30.95 ± 3.34	24.01 ± 1.87	32.11 ± 5.47
(WC) (cm)	98.73 ± 1.73	111.22 ± 6.86	84.25 ± 1.86	105.04 ± 10.81
T CHL (mmol/l)	4.87 ± 1.09	4.97 ± 0.84	5.15 ± 0.91	5.14 ± 1.05
LDL-c (mmol/l)	1.46 ± 0.49	1.37 ± 0.38	1.48 ± 0.42	1.54 ± 0.63
TG (mmol/l)	1.49 ± 0.67	1.96 ± 1.28	1.05 ± 0.41	1.48 ± 0.77
CRP (mg/l)	2.37 ± 2.39	3.94 ± 5.03	1.62 ± 2.08	3.31 ± 3.48
HbA1C (%)	7.09 ± 1.55	7.16 ± 2.24	5.67 ± 0.89	6.60 ± 1.66
T2D (%)	8 (35.0%)	16 (35.5%)	0.00 (0.0%)	69 (25.4%)
HBP (%)	6 (22.0%)	11(24.4%)	1 (5.0%)	102 (37.6%)

*BMI (kg/m*^*2*^*)* body mass index, *WC (cm)* waist circumference, *T-CHL (mmol/l)* total cholesterol, *LDL-c (mmol/l)* low density lipoprotein, *TG (mmol/l)* triglycerides, *CRP (mg/l)* C-reactive protein, *HbA1c (%)* glycated haemoglobin, *T2D* type 2 diabetes, *HBP* high blood pressure.

**Figure 1:**
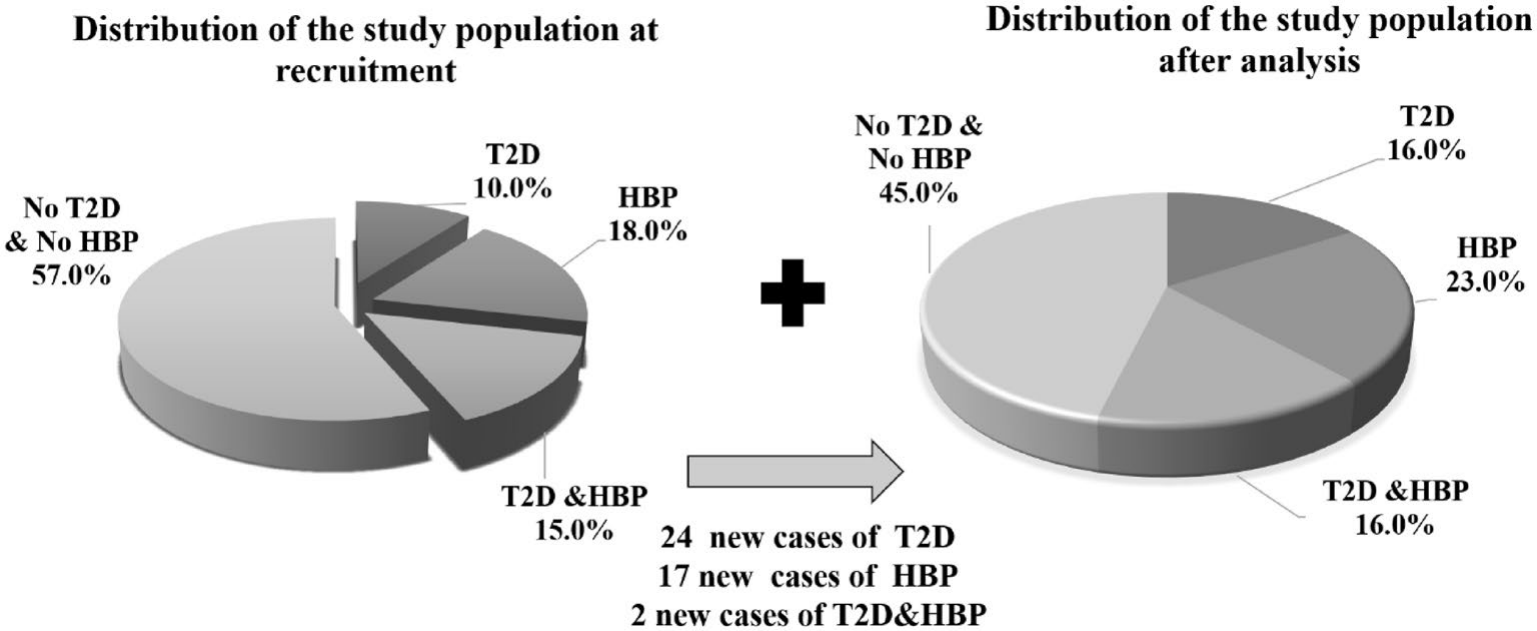
Population status at the recruitment and after clinical examination and biological analysis. *T2D* type 2 diabetes, *HBP* high blood pressure.

Biochemical analyses allowed the discovery of 24, 17 and 2 new cases of T2D, HBP and T2D&HBP respectively. Indeed, at the time of the recruitment, some volunteers were unaware about their diabetes and/or arterial hypertension and were classified as non-diabetics and/or non-hypertensives participants. Taking into consideration the number of known and newly identified cases, we calculated the prevalence of T2D and HBP in the Zaghouan region. Altogether, the study population was composed of 16.0% of diabetes, 23.0% of hypertension, 16.0% of diabetes and hypertension and only 45.0% of the population had neither diabetes nor hypertension (Fig. 1).

**Figure 2:**
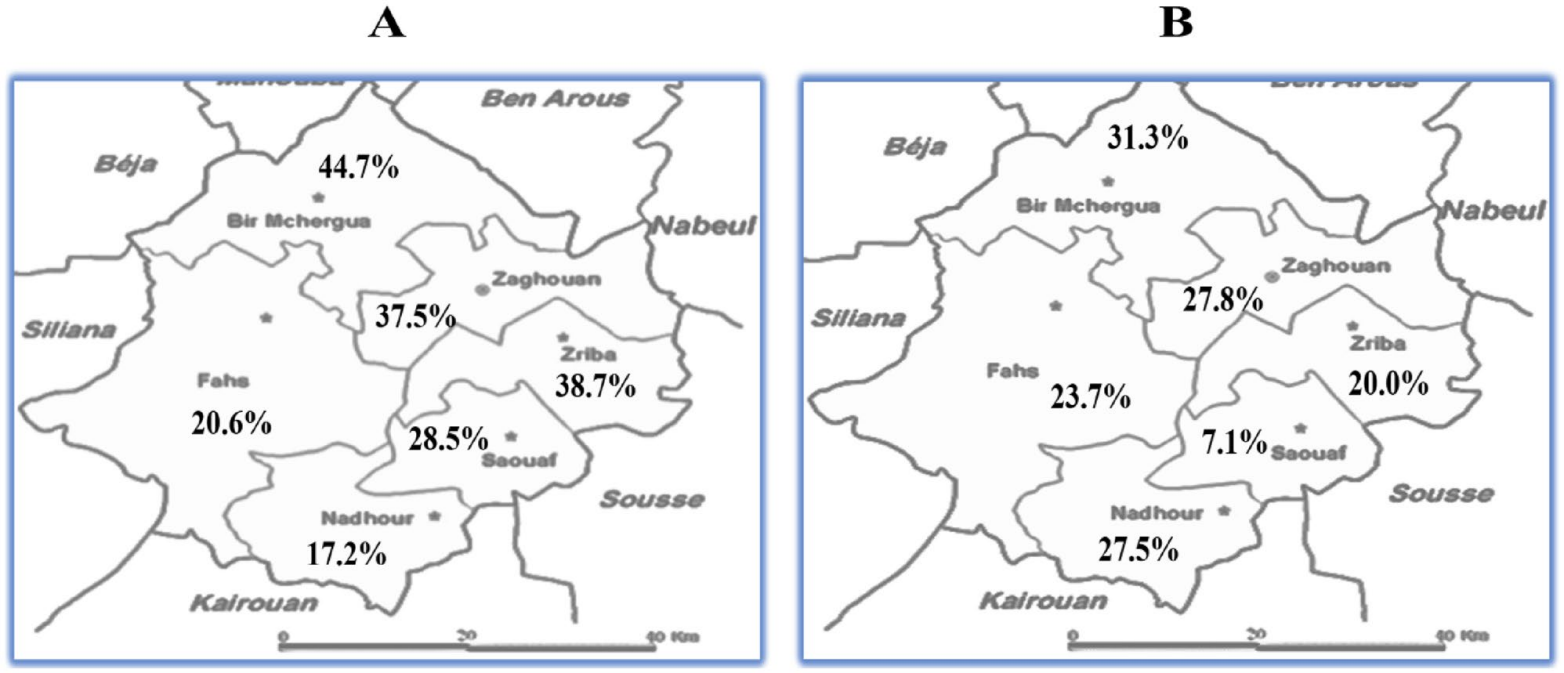
Prevalence of HBP (**A**) and T2D (**B**) in Zaghouan. *HBP* high blood pressure, *T2D* type 2 diabetes.

The overall prevalence of obesity, HBP and T2D among Zaghouan population were at 50.0% (CI 95.0%), 39.0% (CI 95.0%) and32.0% (CI 95.0%) respectively. Diabetes and hypertension proportions were statistically different between Zaghouan districts. We noted that the district of BirMchergua was the most affected by diabetes (31.3%) and hypertension (44.7%) (Fig. 2).

**Figure 3:**
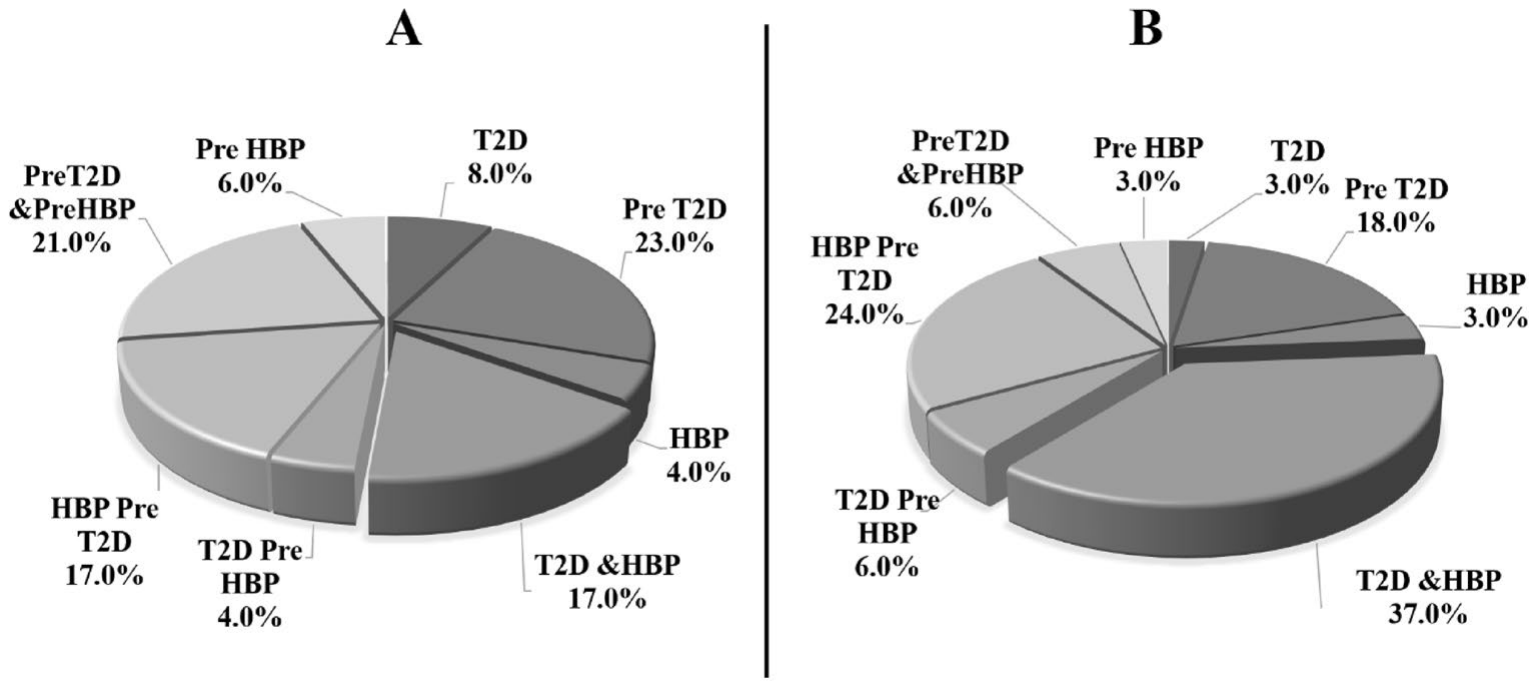
Status distribution among overweight and obese groups. (**A**) Status distribution in overweight group, (**B**) status distribution in obese group. *T2D* Type 2 Diabetes, *HBP* high blood pressure, *Pre T2D* prediabetes, *PreHBP* prehypertension.

In this study, we determined the status distribution of T2D and HBP among overweight (Fig. 3A) and obese (Fig. 3B) groups. Results showed that both diabetes and hypertension were present in 37.0% of the obese group regarding only 17.0% among overweight group.

**Figure 4:**
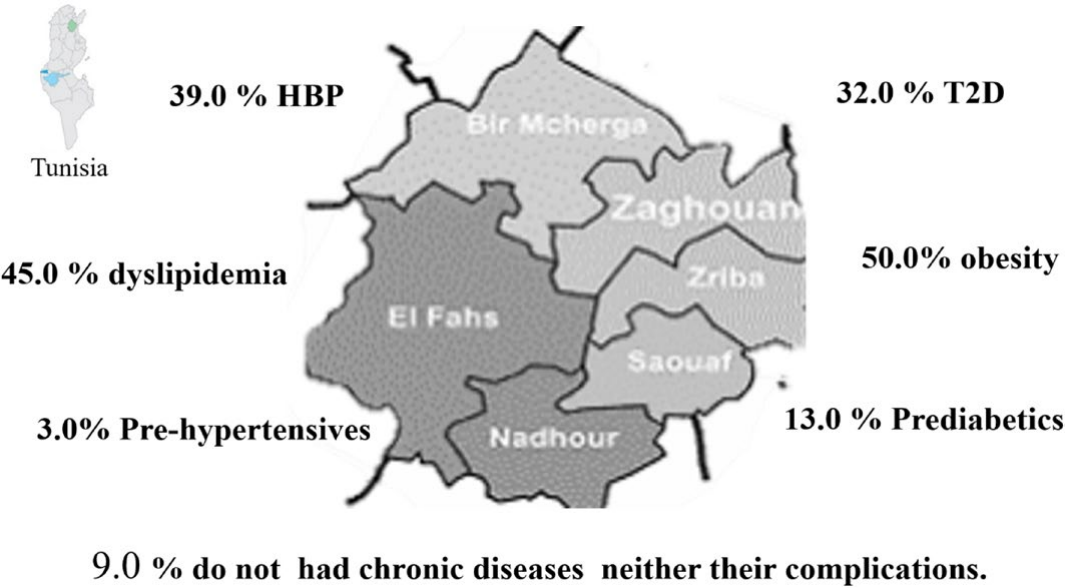
Epidemiological data of chronic diseases in Zaghouan. *HBP* high blood pressure, *T2D* type 2 diabetes.

Regarding diabetes and hypertension complications, more than 60.0% of the diabetic and hypertensive groups of our study population had macro and microvascular complications. About 40.0% of these patients had concomitant dyslipidemia. Our analysis showed that the occurrence of complications was often more than doubled among diabetic and hypertensive patients with concomitant condition such as obesity. Indeed, among diabetic and hypertensive patients with obesity, more than 45.0% had both macro and microvascular complications. Our examination showed that only 9.0% of participants were healthy, non-diabetic, non-hypertensive and non-obese without complications. To sum up, we established an epidemiological map of the Zaghouan region showing the clinical heterogeneity of Zaghouan population regarding chronic diseases (Fig. 4).

### Factors associated with T2D and HBP

The bivariate and multivariate logistic regression analysis conducted between groups with and without diabetes and hypertension showed a significant association of age, BMI, WC, academic level and socio-professional categories with T2D and HBP. Regarding variables related to life-style, there was a strong association between physical activity (OR 5.64, p-value < 0.01), Smoking (OR 4.00, p-value < 0.05), alcoholism (OR 3.00, p-value < 0.05) and both diseases. For biochemical measures; albuminuria, cr**e**atinine and urea showed a statistically significant association with T2D and HBP. Our analysis showed no significant link between 25-OH Vit D and both diseases (Table 4).

**Table 4:** Logistic regression of variables associated with T2D and HBP.

Variables	*p*-value	OR (95% CI)
Age (years)	< 0.01*	0.91 (0.88–0.94)
Gender		
Male	0.5	1.40 (0.58–1.60)
Female		
Academic level		
Primary	< 0.01*	1.75 (1.41–1.08)
Secondary		
University		
Professional		
Socio-professional categories		
Executive and higher intellectual professions	< 0.01	4.17 (0.88–1.37)
Employee		
Worker		
Physical activity		
No	< 0.01*	5.64 (1.07–1.77)
Yes		
Smoking		
No	< 0.05	4.00 (0.56–1.46)
Yes		
Alcoholism		
No	< 0.05	3.00 (1.50–2.44)
Yes		
BMI (kg/m^2^)	< 0.01*	0.91 (0.86–0.95)
WC (cm)	< 0.01*	0.96 (0.94–0.98)
Albuminuria (mg/l)	< 0.05	0.97 (0.94–0.99)
T-CHL (mmol/l)	0.056	1.33 (1.00–1.78)
Creatinine (µmol/l)	< 0.01*	0.98 (0.96–0.99)
LDL-c (mmol/l)	0.056	1.93 (1.00–1.86)
TG (mmol/l)	0.070	0.76 (0.57–1.03)
Urea (mmol/l)	< 0.01*	0.63 (0.51–0.75)
CRP (mg/l)	> 0.9	1.00 (0.98–1.04)
25-OH Vit D (ng/ml)	0.2	1.04 (0.99–1.12)

*OR* odds ratio, *95% CI* 95 confidence interval, *BMI* body mass index, *WC* waist circumference, *T-CHL (mmol/l)* total cholesterol, *LDL-c (mmol/l)* low density lipoprotein, *TG (mmol/l)* triglycerides, *CRP (mg/l)* C-reactive protein, *25-OH Vit D (ng/ml)* Vitamin D, p-value.

*Statically significant (p-value < 0.05).

## Discussion

This study sought to determine the prevalence of T2D and HBP in the governorate of Zaghouan in Tunisia (North East province) as well as their associated risk factors. As far as we know, this was the first study in the Zaghouan region that allowed to have important data and insight on the epidemiology of T2D and HBP in this region. Our findings showed that T2D prevalence in Zaghouan region is higher than that determined in the Governorate of Nabeul (P = 7.2%) (North Eastern) in 2002^20^. In the neighboring region, we found Algeria that has conducted similar studies to determine the prevalence of diabetes or hypertension at the regional level, such as the areas of Tlemcen^21^ and Blida^22^ in Northern Algeria and the El-Menia oasis in central Algeria Sahara^23^. Several studies of this type have also been carried out in the Middle East, including those by Al Khatam Nazih et al. in Dammam and Qatif^24^ in the Eastern province of Saudi Arabia and in Ahvaz in the Southwest of Iran^25^.

Our exploratory study highlighted a high prevalence of diabetes (32.0%, CI 95.0%) and hypertension (39.0%, CI 95.0%) among Zaghouan adults aged of 57.22 ± 13.52 years. Prevalence distribution was statistically different across Zaghouan districts where BirMchergua had the highest prevalence of both T2D (31.3%) and HBP (44.7%). In fact, the high rates of diabetes and hypertension in the district of BirMchergua compared to other Zaghouan’s district may be explained by cultural traditions, socio-economic indicators and environmental factors including water and climate. According to the literature, the district of BirMchergua has a low socio-economic level with a poverty rate of 17.0% and 19.8% unemployment. Also, poor access to health care facilities was noted in the Bir Mchergua area, with only one basic health care center available in the region^26^. The Table 5 summarized the number of hospitals in the six districts of Zaghouan. In addition, the high salinity of the drinking water in the region of BirMchergua^27^ may affects the endocrine system of inhabitants and leads to the development of diabetes and hypertension via sodium-retention mechanism^28,29^.

**Table 5:** The distribution of health care facilities in the six districts of Zaghouan.

District name	Number of hospitals	Number of primary care center
Zaghouan	1	6
Ezzriba	0	7
BirMchergua	0	8
Fahs	0	13
Nadhour	1	7
Saouef	0	4
Total	2	45

The prevalence of diabetes in Zaghouan (32.0%; CI 95.0%) is higher than that reported in entire Tunisia (23.0%). Indeed, in a previous study, the prevalence of diabetes was calculated on a representative sample of the entire Tunisian population from the 24 districts of Tunisia^7^. However, in the present study we included only inhabitants of Zaghouan. The difference in the prevalence may be also explained by the number and the access to health care facilities which is easier in some Tunisian districts such as Tunis (the capital), than in Zaghouan region. For example, 22 hospitals are available in the district of Tunis Vs only 2 hospitals in the Zaghouan region. Compared to neighboring countries such as Algeria, Morocco and Libya, epidemiological situation seems to be the same as observed in Tunisia from 2000 to 2019^7^. This similarity at epidemiological level can be explained by the fact that the Maghreb region shares the same lifestyle and eating habits^30^. Looking at the entire North Africa region, Egypt had the highest prevalence of T2D in 2019. Nevertheless, no data on the prevalence of diabetes in other north African countries have been recorded such as Mauritania^31^. On the other hand the situation of HBP in the Zaghouan region is alarming (P = 39.0%, CI 95.0%) as observed in the whole Tunisia (P = 47.4%, CI 95.0%)^8^.

Our investigation revealed that overweight (30.0%) and obesity (50.0%) were the most emergent public health problems facing the governorate of Zaghouan. This observation is confirmed by epidemiological studies showing a rapid increase in obesity among Tunisian population; it went from 12.2% (6.1% in men, 18.3% in women, p < 0.001) in 2001^32^ to 27.1% in 2016^33^. Our results showed that overweight and obesity were higher among Zaghouan women than Zaghouan men. This observation is in accordance with previous studies highlighting the gender-obesity relationship due to gender differences in cultural (regional) habits^34,35^.

In addition, our study showed that abdominal obesity was also a serious public health condition in the region especially among women (85.0%) and even among men (44.5%). Furthermore, 23.7% and 35.4% of women with diabetes and hypertension respectively had abdominal overweight and obesity. Similarly, 35.3% and 25.0% of men with diabetes and hypertension respectively had abdominal overweight and obesity. Added to that, diabetes and hypertension were present twice as many among the obese group (47.0%) than the overweight one (15.0%). All these results confirm well that obesity is a major risk factor for diabetes and HBP^18,36^.

Moreover, it is interesting to mention that 6.0% of diabetics and 5.0% of hypertensives people were newly discovered after biological analyses. In the same way, Ben Romdhane et al. have identified 7.7% new cases of T2D during a national health examination in Tunisia^10^. This survey showed that 3.0% and 13.0% of Zaghouan adults were respectively pre-hypertensive and pre-diabetic, which will increase the prevalence of these two diseases in Zaghouan population in the next few years.

For cardiovascular complications, results showed that more than 60.0% of diabetic and hypertensive participants had macro and microvascular complications. Indeed, 45.0% of Zaghouan population suffered from dyslipidemia leading to serious complications, including cardiovascular damages^37^. At physiological level, dyslipidemia associated to diabetes promoted toxic changes (oxidation, glycosylation) and increases significantly the risk of cardiovascular diseases^38^. These results are consistent with previous Tunisian studies highlighting high cardiovascular risk among diabetic, obese, and hypertensive patients^39^.

Regarding associated risk factors, our analysis showed a significant association of age, socio-professional categories and academic level with both diabetes and hypertension. These findings are in agreement with the results of the Tunisian Health examination survey^40^. However, early epidemiological Tunisian studies conducted on a random sample from all over Tunisia, showed that employment or education level did not appear to affect the prevalence of T2D^10^. This discrepancy may suggest that geographic regions can play an important role in the spread of diabetes and its risk factors. Moreover, our logistic regression analysis revealed a significant association of alcoholism, smoking and physical activity with T2D and HBP. In particular, the strongest association was found between both diseases and physical activity (OR 5.64, p-value < 0.01). Nevertheless, Ryan et al. demonstrated that heavy smoking has the strongest association with the risk to develop chronic diseases^11^. For biochemical parameters, our investigation revealed an association between Micro Alb, Cr**e**atinine, Urea and both diseases. In this context, Liu et al. showed that TG, and T-CHL were some of the main factors associated to T2D^41^. Our results revealed no significant link between 25-OH Vit D (OR 1.04, p-value = 0.2) and both diseases. This finding is in accordance with a genome wide association study proving the absence of any significant association between variants among Vitamin D related genes neither its metabolites and T2D^42^. Concerning the role of Vitamin D in blood pressure regulation, Grundmann et al. disconfirm any positive correlation^43^. While, meta-analysis studies have shown an inverse relationship between 25-OH Vit D levels and T2D^44^ as well as blood pressure^45^ and justify the use of Vitamin D supplementation for the prevention of T2D^46^ and HBP^47^. Disagreement regarding association between 25-OH Vit D and risk for chronic diseases is exiting and can be explained by heterogeneity among different populations, the study design and sample size.

Furthermore, our results showed a Vitamin D deficiency among Zaghouan population and especially among individuals aged over 70 years old. These results fits with what was reported by Fakhfakh et al. that prevalence of Vitamin D deficiency is common in Tunisia^48^.

Our results could be of great help for health care providers to reduce the incidence of these metabolic diseases and related complications. It seems that the lack of awareness and the limited health education of Zaghouan population is the main cause of rise in diabetes and hypertension prevalence. Therefore, we suggest (i) to promote more education about diabetes, hypertension and obesity, (ii) to develop wide screening for general population and reduce some controllable risk factors such as alcoholism and smoking, (iii) to encourage people to do physical exercises^49^ and (iv) to improve the management of patients with diabetes and hypertension including co-morbidities such as cardiovascular complications^50^. Given its great socio-economic impact, this study could be Conducted at a larger scale in all regions of Tunisia in order to determine the overall prevalence of diabetes and hypertension.

We noticed that the short period of the study cannot support conclusions on the risk of disease, nor on causal relationships and may be considered as a limitation of our study.

To the best of our knowledge, this study is the first of its kind determining the epidemiological situation of chronic diseases among the northern East Tunisian population. It provides data regarding the prevalence of diabetes, hypertension and obesity as well as their associated risk factors. Thanks to this study, we were able to identify new cases of diabetes and hypertension. Also, screening for prediabetic and pre-hypertensive individuals provides new insights towards non-communicable disease prevention. In fact, our results would help to establish a National Program of NCD Prevention adapted to the specificities of the Tunisian population. Taking note, these findings will give valuable information to health policy-makers in the implementation of hygienic and dietary measures.

The originality of this study lies in the heterogeneous genetic landscape of the studied population and the involvement of the CSO. Involving local communities might help decelerate the growing menace of diabetes and hypertension in the country. In addition, involving all the actors and stakeholders i.e. local decision makers, health professionals and citizens on road mapping and public health design will help to bridge the know-do-gap in public health innovation and scientific knowledge sharing. The systematic collaboration of different partners in different sectors allows us to continue networking as an element of sustainability after the end of the project.

## Conclusion

The present exploratory study showed a high prevalence of obesity, hypertension and diabetes in the governorate of Zaghouan and point up the high prevalence of undiagnosed people. We highlighted also an inter-regional difference in terms of T2D and HBP prevalence as well as the associated risk factors like socio-economic features and biochemical parameters with the development of these chronic diseases. We showed that physical activity was the main associated risk factor in the development of T2D and HBP in this region. In perspective, it would be interesting to complete this study with genetic and microbiota investigations in order to better understand the relationship between the two diseases and the gene-environment interaction.

## Data Availability

All data generated or analyzed during this study are included in this published article. For any request regarding the data published in the present paper, please contact the corresponding author Pr. Rym KEFI (rym.kefi@pasteur.utm.tn; rym.kefi@pasteur.tn).
